# Characterization of MCF-12A cell phenotype, response to estrogens, and growth in 3D

**DOI:** 10.1186/s12935-018-0534-y

**Published:** 2018-03-20

**Authors:** Michael F. Sweeney, Carlos Sonnenschein, Ana M. Soto

**Affiliations:** 1 Sackler School of Graduate Biomedical Sciences, Tufts University, 136 Harrison Avenue, Boston, MA 02111 USA; 2 Tufts University School of Medicine, 136 Harrison Avenue, Boston, MA 02111 USA

**Keywords:** Estrogen response, 3D culture, Tissue morphogenesis

## Abstract

**Background:**

Three-dimensional cultures of mammary epithelial cells allow for biologically-relevant studies of the development of the mammary gland in rodents and humans under normal and pathological conditions, like carcinogenesis. Under these conditions, mammotropic hormones play significant roles in tissue morphogenesis. Therefore, a system that recreates the normal, hormonally responsive epithelium would be a valuable tool to study the normal state and its transition to carcinogenesis. MCF-12A cells have been claimed to be non-tumorigenic mammary epithelial cells with reported sensitivity to estrogens. In this study, we aimed at characterizing MCF-12A cells for use in a hormone-responsive 3D culture system to determine their usefulness as a tool to identify normal and abnormal microenvironmental cues.

**Methods:**

MCF-12A cells were single-cell cloned in order to investigate their heterogeneous makeup. The parental cells were then treated with estradiol to investigate proliferative and transcriptional responses through the estrogen receptor alpha. Finally, parental cells and epithelial-like cell-derived clones were seeded in rat-tail collagen I to profile the morphogenesis of multicellular 3D structures. The resultant structures were then analyzed using unsupervised morphometric analysis.

**Results:**

MCF-12A cells consist of epithelial-like colonies which shed elongated, freely growing cells on the colony’s edges. The cells express E-cadherin as well as mesenchymal vimentin but do not express markers associated with myoepithelial cells or fibroblasts. Treatment with estradiol does not affect either the proliferation rate or the induction of gene expression in MCF-12A cells. Parental MCF-12A cells form acini, solid spheres and elongated branching ducts when grown in rat-tail collagen type I matrix, the geometries and distribution of which are altered following the removal of fibroblast-like cells.

**Conclusions:**

MCF-12A cells are a heterogeneous pseudo-epithelial cell line capable of forming a variety of multicellular structures in 3D culture. We found no indication that the cells display estrogen-responsive characteristics, thus refuting previous studies which reported estrogen responsiveness. We report that MCF-12A cells are not suited for use in studies in which differential behaviors of “normal” and “cancerous” estrogen-responsive cells are to be compared.

## Background

Three-dimensional (3D) culture of breast epithelial cells has become a widely accepted and highly relevant tool for the examination of mammary gland biology [1, 2]. Immortalized breast epithelial cells in a 3D context have contributed to a deeper understanding of breast morphogenesis including the role of its microenvironment. In this latter regard, the composition of extracellular matrix [3–5], its stiffness [6, 7], as well as collagen fiber density and organization [4, 7] are all relevant factors responsible for the behavior of individual cells, their intercellular communication, and their organization into complex multicellular structures.

From puberty onward, mammary gland structure and function are dependent on circulating hormones. Curiously, however, most mammary epithelial morphogenesis has been studied using hormone receptor-negative cell lines, such as MCF-10A. During the last decades, a number of immortalized mouse and tumorigenic human-derived mammary epithelial cell lines that express estrogen receptor alpha (ER) and display hormone sensitivity have been established and used for such a purpose; they include HC11 (mouse), MCF7, BT-474 ZR-75-B, MDA-MB-361 and T47D [8, 9]. Cells of the latter established line have recently been shown to form normal-like structures in 3D culture in response to mammotropic hormones. Exposure to estradiol alone resulted in the formation of multicellular structures while further addition of promegestone (progesterone analog) or prolactin resulted in flattened branching structures and budding structures, respectively [10]. While searching for a model that would closely resemble the human mammary tissue, we chose to test MCF-12A cells, a human mammary cell line allegedly reported to be non-tumorigenic and estrogen-responsive.

MCF-12A cells originally established at the Michigan Cancer Foundation are non-tumorigenic human cells that were derived from the reduction mammoplasty of a postmenopausal 63-year-old nulliparous woman [11]. Tissues excised from the donor’s breast revealed non-malignant fibrocystic disease containing intraductal hyperplasia abutted by dense stroma. After they were dissociated, these cells were plated for long-term culture in DMEM/H containing < 0.06 mM calcium, later supplemented with 5% Chelex-treated equine serum and maintained for 1717 days. At this point, “passages one to 15 [were] exposed to 45 °C for as long as 72 h” due to an incubator malfunction. As a result of this event, most cells died. The few surviving cells were expanded and over the next 2 months sublines MCF-12A (adherent cells) and MCF-12F (floating cells) were maintained separately and became “established” [12].

During their initial characterization, MCF-12A cells were implanted subcutaneously in athymic mice, some of which were also implanted with pellets containing 17β-estradiol (E2) but no tumors developed at the inoculation sites in either groups of mice [11]. Given their origin and this outcome, the cell line was labeled as “normal”. Like MCF-10A cells, and unlike tumorigenic MCF7 cells, MCF-12A cells were also considered as ER-negative and non-tumorigenic. Later, Zeillinger [13] described the expression of ER transcripts in MCF-12A as “extremely weak” and Subik [14] was not able to identify any ER positive MCF-12A cells via immunohistochemistry. At least three other studies done using MCF-12A cells described them as ER negative [15–17]. Notwithstanding, literature identifying MCF-12A cells as ER-positive gradually accrued [18–27]. In sum, diverse groups using PCR, western blots, and immunostaining have reached conflicting conclusions regarding the ER status of these cells.

Marchese et al. [28] grew MCF-12A cells in a Matrigel-based 3D model that resulted in the formation of acini and went on to show alterations in lumen formation following treatment with a variety of different estrogens. Western blots purportedly showed the expression of ER and ER-beta proteins by MCF-12A, and MCF7 cells. In addition, estradiol was reported to induce progesterone receptor (PGR) and pS2 in these cells; however, MCF-10A cells were used as a control in this experiment instead of the estrogen-responsive MCF7 cells. This more recent report suggested that MCF-12A cells were a desirable tool for the study of hormone-mediated epithelial morphogenesis.

In order to test the worthiness of the MCF-12A cell line to study hormone-mediated epithelial morphogenesis, we ran experiments from which we conclude that (a) that MCF-12A cells are not responsive to estrogens, (b) the heterogeneous morphology of MCF-12A cells is due to the expansion of tightly growing epithelial colonies which gradually release populations of motile cells that generate morphologically distinct subclones, and (c) when grown in a rat-tail collagen type I matrix, MCF-12A cells produce acini and ducts, resembling those seen in the human mammary gland. Implications of these data are further discussed below.

## Methods

### Cell maintenance

MCF-12A (CRL-10782) and MCF-10A (CRL-10317) cells were purchased from the American Type Culture Collection (ATCC, Manassas, VA) at passage 58. As indicated by the ATCC catalog, cells were grown in Dulbecco’s Modified Eagle’s/F12 medium (DMEM/F12, 1:1) containing 5% equine serum (ES), 20 ng/mL epidermal growth factor, 0.5 μg/mL hydrocortisone, 0.1 μg/mL cholera toxin, and 10 μg/mL insulin. MCF-12A cells were passaged at < 70% confluence, using a 0.25% (w/v) trypsin-0.03% (w/v) EDTA solution; media was replaced every 2–3 days. In our lab, MCF7 cells were grown in DMEM supplemented with 5% fetal bovine serum (FBS). For experiments intended to explore estrogenic effects in 2D culture we used phenol red-free DMEM/F12 (1:1) containing charcoal-stripped serum which lacks endogenous estrogens. Serum was stripped using 5% charcoal-0.5% Dextran T70 (CD) at 37 °C for 1 h. Stripped fetal bovine (CD-FBS) or equine (CD-ES) serum was used for MCF7 and MCF-12A, respectively. Media formulations are listed in Table 1. All cells were maintained at 37 °C with humidity and 6% CO_2_.

**Table 1:** Cell culture media preparations

Name	Base media	Serum	Additives
MCF-12A/MCF-10A	DMEM/F12, 1:1	5% equine	20 ng/mL EGF, 0.5 μg/mL hydrocortisone, 0.1 μg/mL cholera toxin, 10 μg/mL insulin
MCF7	DMEM	5% fetal bovine	
Rinse	DMEM/F12, 1:1, phenol red-free		
CD-FBS	DMEM/F12, 1:1, phenol red-free	5% charcoal–dextran stripped fetal bovine	
CD-ES	DMEM/F12, 1:1, phenol red-free	5% charcoal–dextran stripped equine	20 ng/mL EGF, 0.5 μg/mL hydrocortisone, 0.1 μg/mL cholera toxin, 10 μg/mL insulin

### Immunocytochemistry

Twenty thousand cells were plated and cultured on Nunc Thermonox plastic coverslips (Thermo Fisher) in a 24-well plate for 72 h, washed twice with phosphate-buffered saline (PBS), and fixed in 4% formalin at room temperature for 15 min. Coverslips were then washed twice with PBS and cells were permeabilized with 0.1% Triton X-100 at RT for 15 min. The coverslips were washed in PBS and then blocked with 1:20 goat serum in 1.5% milk for 60 min at RT. Primary antibodies were diluted in BC-11 (0.02 M NaPO_4_H_2_, 0.15 M NaCl, 0.02% Sodium Azide, 1% BSA in distilled water) buffer and diluted antibody (Table 2) was pipetted onto parafilm before the inverted coverslips were placed on top and incubated overnight in a humid chamber at 4 °C. Coverslips were washed twice with PBS, incubated for 1 h with appropriate peroxidase-conjugated secondary antibody diluted in 1% Bovine Serum Albumin (BSA), and visualized with 3,3′-diaminobenzidine (DAB; Sigma) after washing with PBS. Finally, coverslips were washed twice with PBS, counter stained with Harris’ hematoxylin, dehydrated, and mounted on glass coverslips with Permount (Fisher Scientific).

**Table 2:** List of antibodies used for immunocytochemistry

	Source	Dilution
Primary antibody		
Mouse anti-E-cadherin	Novocastra	1:75
Mouse anti-vimentin	Novocastra	1:50
Mouse anti-pankeratin	Sigma	1:500
Rabbit anti-smooth muscle actin	Abcam	1:400
Mouse anti-p63	Santa-Cruz	1:200
Mouse anti-beta-catenin	BD	1:500
Secondary antibody		
Goat anti-mouse peroxidase	Pierce	1:1000
Goat anti-rabbit peroxidase	Pierce	1:1000

### Single cell cloning

MCF-12A cells were trypsinized and spun down at 1200 rpm for 3 min on a bench-top centrifuge and the resulting cell pellet was resuspended in 5 mL of medium. Cells were then counted using a Beckman Coulter Z1 particle counter and diluted to 10 cells/mL. The diluted cell suspension was plated at 100 µL per well in 96-well plates and visually scored for single cell containing wells 24-h later. Wells were assessed for different cell types and eventually expanded serially in 24-, 12- and 6-well plates.

### Dose–response to estradiol (E2)

E2 (Sigma) was diluted in ethanol at a concentration of 1 mM and stored at − 20 °C. MCF-12A cells between passages 61–66 and MCF7 cells between passages 116–131 were used. Cells were plated at a density of 25,000 cells per well in 12-well plates. The next day, cells were rinsed with phenol red-free DMEM:F12 which was then replaced with phenol red-free medium plus 5% CD-FBS (MCF7) or 5% CD-ES (MCF-12A) containing five concentrations of E2 (1 pM, 0.01 nM, 0.1 nM, 1 nM, 10 nM). No E2 was added to control wells. After 5 days, cells were fixed with ice-cold 10% tricholoracetic acid and stained with sulforhodamine B (SRB). Extra dye was rinsed with 1% acetic acid then retrieved using basic Tris buffer (pH 10.5) and read at 515 nm absorbance [29].

### Estrogen-regulated gene induction assays

MCF-12A and MCF7 cells were counted and then plated in 6-well plates at a density of 300,000 cells per well. After 24 h, medium was removed and cells were rinsed once with rinse medium and then incubated in CD-ES or CD-FBS for an additional 24 h. E2, diluted to 0.1, 1, or 10 nM in applicable media (Table 1), was added to cells and cells were incubated at 37 °C for 48 h. Cells were then rinsed once with sterile PBS before being lysed according to the Qiagen RNeasy protocol. RNA was isolated and quantified with a Nanodrop photospectrometer and then 2 μg of RNA was used to prepare cDNA libraries using Superscript reverse transcriptase (Invitrogen). Estrogen-responsive transcripts were quantified via qRT-PCR using a SYBR green master mix (Bio-Rad) in an iQ5 thermo cycler. Fold change induction was calculated with the Bio-Rad software (version 2.1) and normalized to the expression of *RPL19* transcripts. Primer sequences are shown in Table 3.

**Table 3:** Estrogen responsive gene induction assay primer sequences

Gene	Forward primer	Reverse primer
L19	5′-TAGTCTGGCTTCAGCTTCCTC-3′	5′-TCTGCAACATCCAGCTACCC-3′
Estrogen receptor alpha	5′-TAAATGCTGCCATGTTCCAA-3′	5′-CCTGTGAGAGAACAGAAACTGG-3′
Amphiregulin	5′-GTGGTGCTGTCGCTCTTGATACTC-3′	5′-TCAAATCCATCAGCACTGTGGTC-3′
Progesterone receptors A/B	5′-GAGGATAGCTCTGAGTCCGAGGA-3′	5′-TTTGCCCTTCAGAAGCGG-3′

### 3D cell culture

3D cultures were generated as previously reported [30]. Briefly, a 1 mg/mL rat-tail collagen type I solution (Corning) was made according to the manufacturer’s “alternate gelation procedure” and stored on ice prior to use. Cells were detached with trypsin, pelleted at 1200 rpm × 3 min and then resuspended in 10 mL of MCF-12A medium and counted. 75,000 cells were seeded per gel per 1.5 mL of collagen solution in a 12-well plate. After 30 min at 37 °C, 2 mL of MCF-12A medium was added to each well and the gel was detached from the edges of the well using a sterile pipette tip. Culture medium was changed every 2–3 days and gels were harvested after 14 days. Gels were processed for paraffin embedding for histological analysis and whole mount microscopy as described in [10]. Gel diameter was measured using Axiovision (Zeiss) imaging software.

### Analysis of epithelial structures

Whole mounted, carmine-stained gels were imaged at 200× with a LSM800 (Zeiss) confocal microscope. A region of interest was established 500 µm inward from the apex of each semicircular gel and maintained for all replicates. An area 120 µm thick was imaged using a HeNe 633 laser. The Zeiss software was used to create arrays of tiles 5X3 wide which were stitched together with a 20% overlap. Stitched images were then analyzed with the “Software for Automated Morphometric Analysis” (SAMA [31]) that allows for the unbiased, unsupervised analysis of physical attributes of each epithelial structure in the region of interest. Raw data produced by SAMA was filtered based on volume (1000 µm^3^ cutoff) and analyzed using Prism Software.

### Statistical analysis

One-way ANOVAs were performed to compare cell proliferative effects of estradiol on MCF7 and MCF12A cells. Dunnett 2-sided t-tests were applied to analyze differences in gene expression data. Students t-tests were used to compare gel contraction. Mann–Whitney non-parametric t-tests were used to analyze 3D morphometric data derived from SAMA.

## Results

### Description of parental cells

**Fig. 1:**
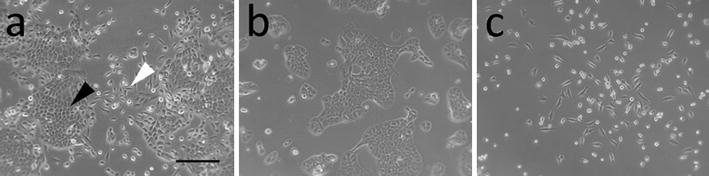
MCF-12A cells grow as a heterogeneous population. Parental cells grow as epithelial plaques surrounded by single fibroblast-like and spherical cells (**a**). Single cell cloning lead to the isolation of epithelial-like colonies (**b**) and a mixed population consisting of both fibroblast-like and spheroid cells (**c**). (Scale bar = 250 µm)

After receiving frozen stocks from ATCC, MCF-12A cells were expanded in their recommended media and passaged twice. Consistent with previous publications, the cells grew as a heterogeneous population [11, 32]. A subpopulation of MCF-12A cells from this initial stock grew as colonies of cobblestone-like cells (Fig. 1a, black arrowhead). The epithelial cells were mononuclear with a well-defined nucleolus and nuclear size varied between cells within the epithelial plaques. Isolated spheroid and elongated fibroblast-like cells were observed beyond the perimeter of the epithelial colonies interspersed with domed cells (Fig. 1a, white arrowhead).

### Single cell cloning observations

Based on the assumption that there were two or three subpopulations of cells within MCF-12A parental cells, single-cell cloning was performed in order to isolate the varied morphological populations. Following expansion of single-cell colonies, cobblestone-like colonies (BF9) were isolated, further expanded and frozen. These colonies contained cells of homogeneous morphology and formed large plaques with well-defined borders (Fig. 1b). After 5 passages, the edges of these colonies began to accumulate elongated cells that migrated away from the plaque. During subsequent passages, these spindle-shaped cells, which briefly appear spheroid following cell division, continued to multiply and populated the spaces in between epithelial plaques. After 10 passages the initial cobblestone-like clonal population reverted to a heterogeneous population resembling the parental cell line.

An additional subpopulation was selected containing only spheroid and elongated cells types (Fig. 1c). Cells in this sub-population appeared highly motile and grew separately without forming colonies. These cloned cells failed to reestablish the cobblestone cell morphology seen in the parental line.

### Epithelial, myoepithelial, and mesenchymal marker expression in parental MCF-12A cells

**Fig. 2:**
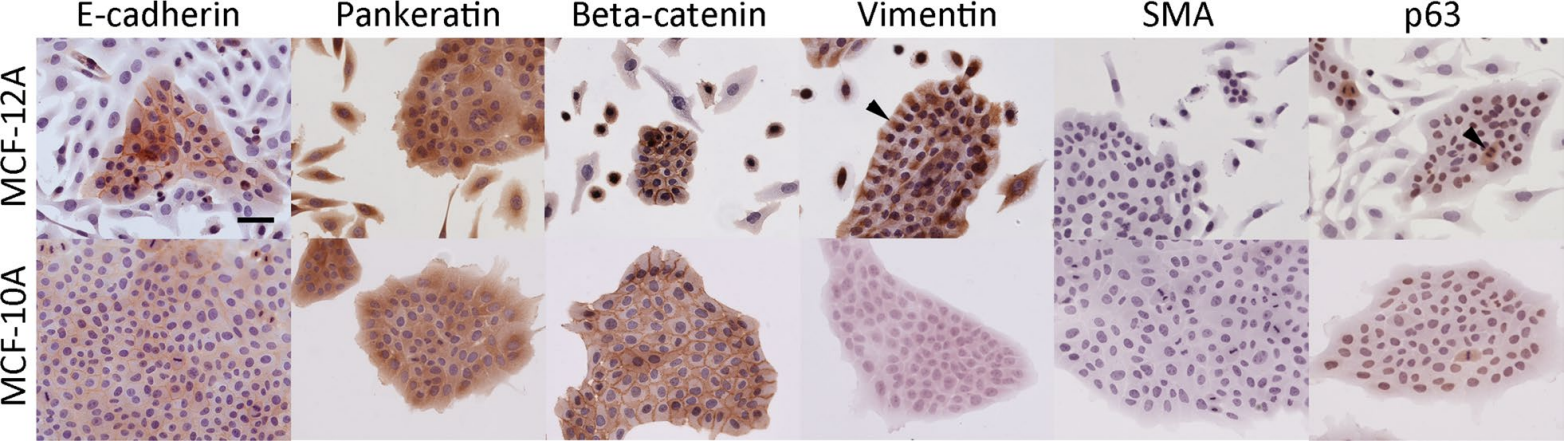
MCF-12A cells express epithelial and mesenchymal cell markers. E-cadherin is expressed throughout plaque-associated cells and highly localized to cell–cell junctions in the center of epithelial plaques. Localization at cell–cell junctions decreases at plaque edges. Cytokeratins are expressed in all MCF-12A subtypes. Beta-catenin is expressed by epithelial-like MCF-12A colonies with increased localization at their cell–cell junctions and dispersed throughout the cytoplasm of fibroblast-like and spheroid cells. Vimentin is expressed in the cytoplasm of all cells but concentrated in fan-like projections from peripheral cells. P63 is expressed in dividing cells but absent from others. MCF-10A cells used as a control for epithelial marker expression. Scale bar = 100 µm

While several publications have alluded to the expression of some epithelial and/or mesenchymal markers by MCF-12A cells, a more thorough analysis of these cells studied under those conditions has yet to be published. MCF-12A cells did not adhere to acid-washed glass coverslips or coverslips coated with collagen or poly-lysine. Cells did, however, adhere to plastic coverslips. To investigate the cell states present in MCF-12A cultures, a panel of epithelial and mesenchymal markers was employed. MCF-10A cells were used as a positive control for epithelial cell markers. The epithelial sub-population of MCF-12A expressed E-cadherin diffusely throughout the cells with darker staining localized to cell–cell junctions (Fig. 2) while their elongated fibroblast-like counterparts failed to express E-cadherin (Fig. 2). Expression of E-cadherin at cell–cell junctions was more pronounced in the center of epithelial plaques and diminished in between peripheral cells. MCF-12A cells also expressed vimentin. Remarkably, cells at the epithelial plaque edges showed accumulations of vimentin radiating away from the plaques center, and vimentin was excluded from areas of cell–cell contact (Fig. 2, arrowhead). Cells separated from epithelial plaques expressed vimentin throughout their cytoplasm. As expected, vimentin expression was absent from control MCF-10A cells.

Cytokeratins were expressed in all MCF-12A cells. Beta-catenin was expressed throughout, though strongly at cell–cell interfaces, but not in the nuclei of cells within epithelial plaques formed by both MCF10A cells and MCF-12A cells. However, MCF-12A cells growing isolated from plaques contained beta-catenin throughout their cytoplasm and in their nucleus in some smaller cells. Despite their appearance, elongated fibroblast-like MCF-12A cells did not express the myoepithelial marker smooth muscle actin (Fig. 2). The myoepithelial cell marker p63 was expressed in the nucleus of epithelial-like MCF-12A cells and in the cytoplasm of dividing cells within the epithelial plaques. This distribution of p63 expression is similar to that seen in colonies of MCF10A cells (Fig. 2, lower right). MCF-12A cells growing away from epithelial plaques, however, lacked expression of p63 in both their nucleus and cytoplasm.

### MCF-12A cells proliferate equally with or without E2

**Fig. 3:**
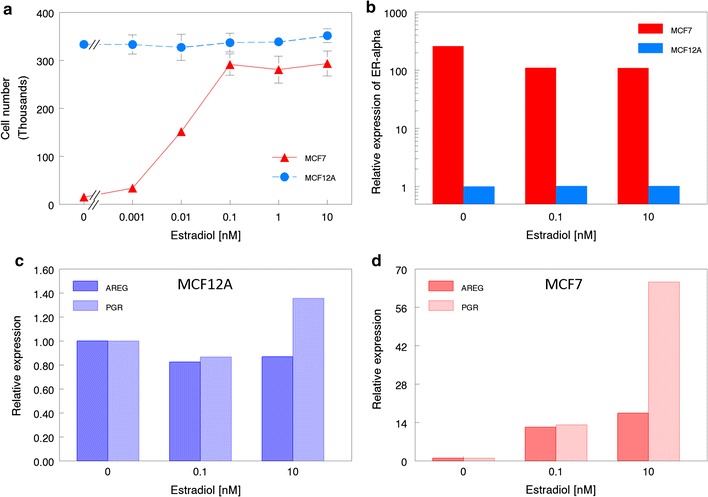
MCF-12A cells do not respond to stimulation by estradiol. MCF-12A cells proliferate maximally under all experimental conditions while MCF7 cell proliferation is inhibited in the absence of estradiol and stimulated at increasing concentrations (**a**). Relative to MCF7 cells, MCF-12A cells do not express estrogen receptor alpha (**b**). The induction of estrogen responsive genes amphiregulin (AREG) and progesterone receptor (PGR) is not seen in MCF-12A (**c**) cells while estrogen receptor alpha-positive MCF7 cells show expression following estradiol exposure (**d**). Note differences in y-axis scales to accommodate variation in expression differences

ER-positive MCF7 and T47D cells have been shown to exhibit a proliferative dose response to E2 when cultured in medium containing CD stripped serum-supplemented medium, and are inhibited from proliferating by a serum-borne inhibitor [33]. This represents a standard case for studying the proliferative effects of E2 [34]. Thus, had MCF-12A cells been shown to be ER-positive, enhanced proliferation of these cells in the presence of estrogens accompanied by quiescence in medium supplemented with CD-stripped serum would have indicated the functionality of the ER. However, as shown in Fig. 3a, while MCF7 cells proliferated maximally in E-stripped serum supplemented with 0.1 nM E2 and did not proliferate under 5% CD-FBS conditions, MCF-12A cells proliferated maximally regardless of whether the cells were exposed to CDFBS or to CDFBS plus E2. These results indicated that MCF-12A cells were not influenced in their proliferative behavior by the presence of CDFBS or of E2 in the culture medium.

### MCF-12A cells lack expression of ER and its transcriptional targets

The proliferative response of MCF7 cells to E2 was accompanied by a decrease in the expression of ER transcripts (Fig. 3b). In CD-FBS media, MCF7 cells expressed ER transcripts ≥ 250-fold higher than MCF-12A cells when maintained in a comparable medium. Increasing concentrations of E2 resulted in decreased expression of ER transcripts by MCF7 cells but had no effect on the expression by MCF-12A cells. We next attempted to determine whether or not there was transcriptional activation by ER in MCF-12A. The expression of progesterone receptor (PGR) and amphiregulin (AREG) by MCF12A cells showed no estrogen-dependent induction (Fig. 3c), unlike that observed in MCF7 cells (Fig. 3d). The y-axis scales highlight the magnitude of transcript expression differences between the cell lines.

### MCF-12A cells form ducts and acini in 3D

**Fig. 4:**
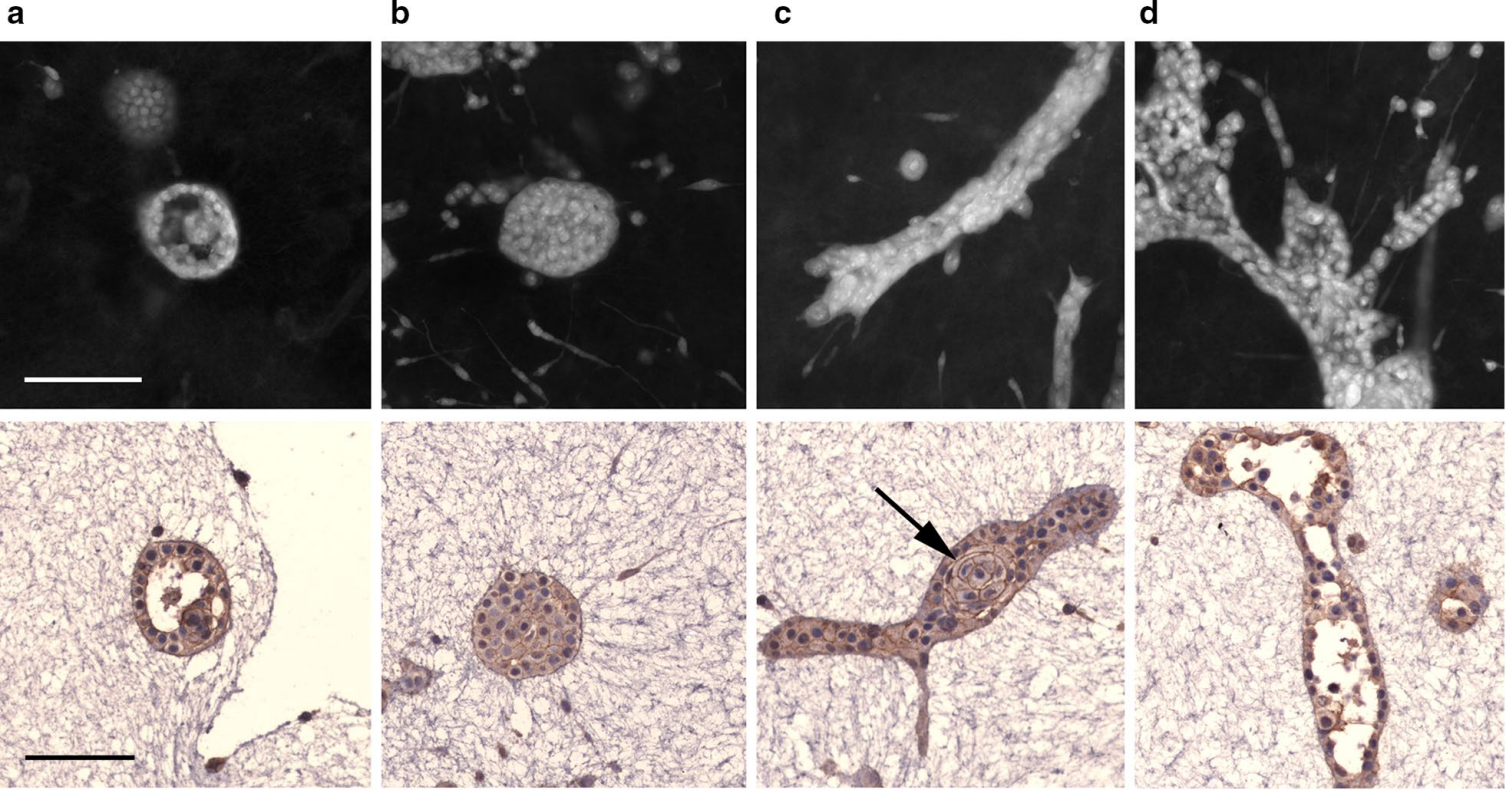
3D growth of MCF-12A cells in rat-tail collagen after 14 days. Confocal projections of select structures (top row) and E-cadherin stained cross sections of corresponding structures (bottom row). MCF-12A cells form hollow (**a**) and solid acini (**b**) as well as solid bifurcating ducts (**c**) and lumenized branching ducts (**d**). Spherical clusters of concentric cells are often seen within elongated ductal structures (black arrow). Scale bar = 100 µm

To study MCF-12A morphogenesis in 3D, we employed an embedded; floating rat-tail collagen type I culture system. When examined 14 days after seeding, MCF-12A cells showed multiple phenotypes. The majority of them were solid spherical structures varying in size (Fig. 4a). Acini with single-cell thick borders and cleared lumena were observed (Fig. 4b). MCF-12A cells also formed branching, ductal structures (Fig. 4c), of which some showed lumen (Fig. 4d). Spherical clusters of concentric cells (Fig. 4c, arrow) were often seen within ductal structures. In addition to multicellular structures, spindle-shaped single cells grew throughout the gel.

**Fig. 5:**
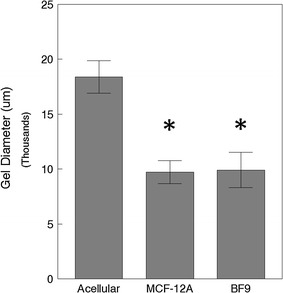
Contraction of collagen gels after 14 days in culture. The addition of MCF-12A cells to rat-tail collagen gels results in a decreased gel diameter compared to acellular gels. MCF-12A-derived BF9 cells cause a similar extent of contraction. Asterisks signify gels that are significantly more contracted (p-value < 0.05) than acellular gels

Addition of parental MCF-12A cells caused collagen gels to contract compared to acellular gels after 14 days (Fig. 5). BF9 cells contracted gels to a similar degree.

### MCF-12A morphogenesis varies due to cell-subtype dynamics

**Fig. 6:**
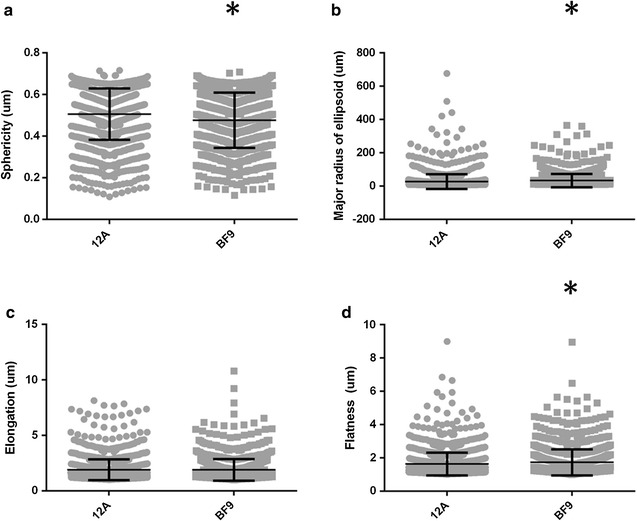
Non-epithelial-like subpopulations alter the morphology of epithelial-like MCF-12A cells. Structures formed by the heterogeneous parental MCF-12A cells are more spherical (**a**), have shorter major radii (**b**), and are flatter (**d**) than those formed by primarily epithelial subclone BF9. Elongation (**c**) is not significantly different between the parental cells and BF9 cells. Structures were pooled from all technical and biological replicates for analysis (MCF-12A n = 1537, BF9 n = 1099). Error bars show SD. Asterisks denote p values < 0.05

Because of the unique 2D phenotypes of the two cellular subtypes isolated from MCF-12A parental cells, we asked whether or not the epithelial-like populations could form different structures in 3D. We were specifically interested in observing complex, multicellular epithelial structures. Therefore, the SAMA data was analyzed with filters in place to ignore single cells and non-relevant entities, such as sheets of cells or gel artifacts. All structures below 1000 µm^3^ were removed prior to statistical analysis. As shown in Fig. 6, structures formed by BF9 cells are longer (p-value ≤ 0.0001) and flatter (p-value = 0.0007) on average than those found in parental cell gels. Conversely, MCF-12A structures were more spherical structures on average (p-value ≤ 0.0001). These parameters were also statistically significantly different at the 500 µm^3^ (except flatness) and 2000 µm^3^ cutoffs (data not shown).

## Discussion

Hormonal regulation of morphogenesis has been explored in a few established culture models that use immortalized cell lines such as MCF7 and T47D [10, 35]. Initially, we were interested in employing the MCF-12A cells in our 3D culture model due to their normal, non-tumorigenic origin and their alleged estrogen sensitivity. To our knowledge, currently, no “normal”, ER-positive established cell lines are available. Claims of estrogen-sensitivity made about MCF-12A cells appealed to us for the exploration of the human mammary gland in both normal development and in pathological contexts, like carcinogenesis. It is acknowledged, however, that a given cell line often varies widely in hormone receptor content from one lab to the next [36–38]. Thus, before embarking in an extended study of the subject it was necessary to accurately characterize the cell line to be used in such a project.

### Morphology and cell-type markers

Unlike MCF-10A cells, which form epithelial plaques with smooth, defined boarders in 2D cultures, MCF-12A cells underwent a process of phenotypic changes during their propagation. Namely, cells at the edge of plaques lost the plasma membrane localization of E-cadherin and beta-catenin, altered their distribution of vimentin, and became highly motile. Morphologically, these cells appeared to be migrating away from the plaques due to their fan-like projections (Fig. 2). A possible explanation for the dynamic morphologies seen in MCF-12A cells would have been the co-expression of E-cadherin and vimentin. An inverse relationship between E-cadherin and vimentin in intact tissues is considered to be an indicator of epithelial–mesenchymal switching [39]. When vimentin was overexpressed in MCF7 cells they adopted mesenchymal morphologies with vimentin localized within the cells in a pattern similar to that seen at the edges of MCF-12A cell colonies [40]. Apparently, the mechanical and biochemical constraints imposed upon MCF-12A cells growing at the center of epithelial plaques are reduced on cells residing in the plaque’s periphery.

MCF-12A cells express gene products that are associated with both luminal and basal subtypes and have features of basal progenitor cells [41]. Dual expression of E-cadherin and vimentin has been linked to highly aggressive tumors in other tissues including those in the lung and neck [42, 43]. However, MCF-12A cells are considered non-invasive [11]. Expression of p63 has been reported to be restricted to the basal layer of complex glands such as the mammary gland and prostate [44, 45]. Decreasing p63 expression in these cells was linked to a need to replenish cells composing the luminal compartment. The finding that p63−/− mice lack stratified epithelium would support this notion [46]. The transient expression of p63 in dividing epithelial-like MCF-12A and MCF-10A cells is likely to affect the behavior of their daughter cells. However, non-epithelial MCF-12A cells do not express p63 (Fig. 2), further questioning the cell type that MCF-12A cells more closely resemble in vivo.

### Estrogenicity

Until now, the estrogen responsive profile of MCF-12A has been ambiguous. In the original report, MCF-12A xenografts failed to grow in the presence of E2 and additional findings, mentioned but not shown by the authors, suggested that the cells may have been ER-negative [11]. The expression of ER is not sufficient to describe a cell line as being responsive to estrogens. Nevertheless, published reports claimed these cells to be ER positive and have therefore been used as normal controls to genuine estrogen-responsive cancer cells. Mischaracterizations such as these lead to contradictory conclusions and may have added to the increasingly poor reproducibility of biomedical studies [47]. By using multiple assays, here we have now shown that MCF-12A cells are unresponsive to E2 in culture conditions, both in regard to the controls of cell proliferation and of the expression of estrogen-regulated genes.

Estrogen responsive cells remain quiescent in the presence of charcoal-stripped serum and the absence of E2 and proliferate in the presence of estrogenic compounds [48]. The proliferative effect of estrogen on these cells only becomes effective when serum supplemented to the basic cell culture medium is CD stripped; in this condition, cells enter proliferative quiescence due to the effect of the serum-borne inhibitor, albumin [33, 49]. Addition of physiological levels of estradiol neutralize the inhibitory effect of CD serum and thus the capacity of these cells to proliferate is restored. Instead, MCF-12A cells proliferated equally well regardless of the media’s estrogen content. This implies that MCF-12A cells are insensitive to CD-serum induced quiescence and thus are non-responsive to both the serum-borne inhibitor and to estradiol.

In order to explore whether the lack of proliferative response to E2 was due to absence of ER, we tested transcriptional activation resulting from estradiol-ER binding to estrogen responsive cells (MCF7) and compared it to that of MCF-12A cells. The binding of estrogenic chemicals to ER affected the induction and/or attenuation of different transcripts within MCF7 cells [50]. We used qRT-PCR to investigate the induction of estrogen-responsive genes in MCF7 cells by adding estradiol concentrations that resulted in maximal proliferative responses. While the expression of both amphiregulin and progesterone receptor was significantly increased in MCF7 cells treated with estradiol, MCF-12A fail to significantly express either gene product following 48 h of estradiol stimulation. These results imply that, in addition to the lack of a proliferative response by estrogens, exposure to estrogens also failed to induce specific gene transcription in MCF-12A cells. Moreover, relative to MCF7 cells, MCF-12A cells express levels of ER transcripts similar to those seen in ER-negative cell lines. The basal-like transcriptional profile of MCF-12A cells further suggests that these cells are ER negative, as only luminal cells express ER in tissues [51].

### Growth in 3D

The formation of epithelial structures in 3D culture allows for the in vitro study and manipulation of the mammary epithelium. We have previously shown that some cell lines form both acini and ducts when grown in collagen with and without the addition of Matrigel [30, 52]. Herein, we examined the capacity of MCF-12A cells to form normal epithelial tissue structures. In a 3D context, the formation of lumena is a hallmark of normal mammary phenotype [5]. When grown on top of laminin-rich extracellular matrix for 4 days, MCF-12A cells formed rounded, non-lumenized structures [41]. Using a similar cell model, these early round structures were shown to form growth-arrested acini consisting of polarized cells after 16 days in culture [28]. After 14 days of being embedded in rat-tail type I collagen, in addition to lumenized acini and solid, round structures, MCF-12A cells in our growth conditions organized into large, lumenized, branching structures. The formation of duct-like structures by MCF-12A cells has previously been described only when grown on top of matrix derived from nulliparous rat mammary glands [53]. This implies that rat-tail type 1 collagen recapitulates the mammary gland environment found in vivo better than in several other 3D models currently in use. The rounded clusters of concentric cells (Fig. 4c, arrow) never occurred outside of other structures implying that their formation is dependent on the interruption of interactions between those cells and the collagen matrix. MCF-10A cells grown in rat-tail type I collagen only form acini and short non-branching ducts without any “rosebud” structures.

### SAMA as a tool to analyze epithelial structures grown in 3D culture

We employed the SAMA package for the unbiased, unsupervised analysis of MCF-12A structures formed in collagen gels. SAMA allows for unsupervised, and hence unbiased and automated measurements of a host of biologically relevant physical parameters of epithelial structures, an ability that is useful in analyzing large number of structures. Another of the advantages of the SAMA software is the ability to filter the data based on both size and biological relevance of the structures analyzed. This software package enabled us to analyze hundreds of structures within a 3D space for geometric parameters that otherwise would have been difficult to perform.

The variability of structural shapes formed in other areas of these gels may be due to the distribution of MCF-12A cells migrating toward the perimeter of each gel. The ability of MCF-12A to drastically contract the gels (Fig. 5) and the heterogeneous collagen fiber organization may be responsible for this variability [30]. Gel contraction of similar magnitude has been previously described when MCF-10A cells were grown in rat-tail collagen and resulted in a non-homogenous distribution of structures [30]. The finding that fibroblast-like cell-depleted BF9 subclone contracted gels at a similar degree to that seen by the parental cell population implies that those non-epithelial-like cells do not contribute to gel contraction. If those cells were responsible for increased gel contraction, assuming their contributions to be additive, BF9 gels would be expected to contract to a degree intermediate between acellular and parental gels.

The results of SAMA analysis confirm that MCF-12A parental cells form spherical, acinar and duct-like structures. Upon removal of the MCF-12A cells which grow away from the epithelial plaques in 2D, the remaining cells form longer, flatter structures than those produced by the heterogeneous parental cell line, on average. The differences seen between parental and BF9 gels are not simply due to the exclusion of individual spindle-shaped cells but, rather, imply an interaction between the two cell types in 3D which results in distinctly different structural compositions. Human fibroblasts have been shown to speed up the morphogenesis of epithelial structures formed by MCF-10A cells and increase the number and length of ducts formed when seeded jointly in collagen gels; this effect is likely due to fibroblast-mediated formation of thicker, parallel collagen fibers [4, 54]. However, the single cell population of MCF-12A acts differently than fibroblasts; this effect might be due to fibroblasts interfering with the ability of the epithelial cells to alter collagen organization leaving thinner, less organized fibers favoring the formation of rounded acinar structures.

## Conclusions

We have documented that MCF-12A cells are a non-tumorigenic, heterogeneous, estrogen receptor-negative cell line that express a combination of epithelial and mesenchymal markers. In floating rat-tail type I collagen gels, MCF-12A cells form complex, lumenized acini and ducts, the characteristics and distribution of which are altered by either the presence or absence of different subpopulations. Based on the time-dependent progressive population changes within the parental cell line and the complex interactions between these two cell types in 3D, MCF-12A cells appear unsuitable for use in epithelial morphogenesis studies when compared to MCF10A cells.

## Abbreviations

AREG: amphiregulin
CD: charcoal dextran
E2: 17-beta estradiol
ER: estrogen receptor alpha
ES: equine serum
FBS: fetal bovine serum
PGR: progesterone receptor
